# The potential key genes within focal adhesion that regulate mesenchymal stem cells osteogenesis or adipogenesis in microgravity related disuse osteoporosis: an integrated analysis

**DOI:** 10.3389/fendo.2025.1469400

**Published:** 2025-03-10

**Authors:** Haoyang Zhao, Xiaolin Tu

**Affiliations:** Laboratory of Skeletal Development and Regeneration, Key Laboratory of Clinical Laboratory Diagnostics (Ministry of Education), College of Laboratory Medicine, Chongqing Medical University, Chongqing, China

**Keywords:** osteoporosis, osteogenesis, adipogenesis, focal adhesions, mesenchymal stem cells

## Abstract

This study aimed to identify key genes related to focal adhesions (FA) and cells involved in osteoblast (OS) and adipocyte (AD) differentiation in osteoporosis. A mouse model of disuse osteoporosis was made by hindlimbs unloading (HLU)/Tail - suspension. Micro - CT and histological analysis were done, and differentially expressed genes (DEGs) from GSE100930 were analyzed. Soft clustering on GSE80614 OS/AD samples found FA - related candidate genes. protein-protein interaction (PPI) network and cytoHubba’s Degree algorithm identified key FA - genes, validated by quantitative polymerase chain reaction (qPCR). Key OS/AD - associated cells were identified by single - cell analysis. The mouse model showed decreased bone density, microstructure damage, increased marrow adiposity, and altered gene expression. Key FA - related genes for osteogenesis (ITGB3, LAMC1, COL6A3, ITGA8, PDGFRB) and adipogenesis (ITGB3, ITGA4, LAMB1, ITGA8, LAMA4) were found and validated. Key cells (chondrocyte, adipocyte, and osteoblast progenitors) are involved in specific pathways, with osteoblast progenitors having stronger interactions. Pseudotime analysis implies differentiation from chondrocyte progenitors to adipocyte, then osteoblast progenitors. This study provides new insights for disuse osteoporosis research.

## 1 Introduction

Osteoporosis is a global systemic bone metabolic disease that currently affects over 200 million people. It primarily increases the risk of fractures in patients by reducing bone density and degenerating the microstructure of bone tissue (1, 2). With the increasing incidence of osteoporosis, it has become a major public health issue globally, imposing a significant burden on social welfare and healthcare systems (3). This warrants sufficient attention and the adoption of comprehensive multidisciplinary prevention and treatment strategies, thereby necessitating urgent exploration of the mechanisms of osteoporosis to identify effective molecular targets for intervention.

Research indicates that the occurrence of osteoporosis is closely related to various factors, including hormonal changes and natural aging. Specifically, mesenchymal stem cells (MSCs) play a central role in disease progression, with the potential to differentiate into various cell types such as osteoblasts and adipocytes (4). The lineage differentiation and fate determination of MSCs are importantly linked to the development of osteoporosis (5–7). Further evidence suggests that changes in estrogen levels (8–10) and age-related primary osteoporosis (11–13) are accompanied by an increase in adipocyte accumulation in the bone marrow cavity, as is the case with disuse osteoporosis (14–17). Thus, regulating the balance of MSC differentiation into osteoblasts or adipocytes has become a potential strategy for preventing or treating osteoporosis.

Focal adhesions (FAs) are key structures for cell interaction with their external environment, not only serving in physical connections but also as crucial hubs for various intracellular and extracellular signaling, including mechanotransduction (18). Through the interaction between integrins and the extracellular matrix (ECM), FAs participate in regulating many biological processes, including cell migration, proliferation, differentiation, and survival (19, 20). Given that the pathogenesis of osteoporosis involves these complex biological processes and the recognized role of focal adhesion genes in cell fate determination and differentiation (21–23), exploring how these genes regulate the differentiation of MSCs into osteoblasts or adipocytes in disuse osteoporosis can deepen our understanding of the disease mechanisms and aid in developing therapeutic strategies based on these targets or genes.

This study aims to analyze the transcriptional data of mesenchymal stem cells related to disuse osteoporosis from public databases (GSE100930) (24), and their osteogenic and adipogenic differentiation time data (GSE80614) (25), exploring the expression characteristics of key focal adhesion-related genes. Key genes were validated using quantitative polymerase chain reaction (qPCR) in bone marrow mesenchymal stem cells from a mouse model of disuse osteoporosis. The study also aims to use bioinformatics techniques to reveal the diagnostic potential and molecular regulatory mechanisms in the osteogenic or adipogenic processes of osteoporosis. Additionally, this research utilized single-cell datasets of mesenchymal stem cells (GSE147287) (26) to analyze the expression characteristics of key genes in various cell clusters in osteoporosis, identifying crucial cell clusters and validating them in animal models through histological examination, providing new directions for further research on disuse osteoporosis.

## 2 Materials and methods

### 2.1 Establishment of disuse model in mice

Toward the end of the last century, NASA and the University of California collaboratively developed a classical mouse model known as the hindlimbs unloading (HLU)/tail-suspension method (27). Designed to mimic disuse osteoporosis in long bones under microgravity conditions, this model effectively simulates the absence of axial stress in terrestrial environments (28–30). For this experiment, male C57BL/6J mice, each 14 weeks old and of a similar weight, were obtained from the Experimental Animal Center of Chongqing Medical University (China, Chongqing). These mice were assigned to either an hindlimbs unloading group (HLU) or a normal loading group (NL). In the HLU group, mice were individually housed in tail-suspension cages with their tails securely fastened to a metal frame using medical tape and cotton threads to evenly distribute the stress. The duration of the HLU experiment was 14 days. Based on the rodent-to-human age conversion, mice aged 14 to 16 weeks are roughly equivalent to two years in the human lifespan (31). Considering the data on bone loss, this model can simulate unloading for over a year, potentially even close to two years (32). Environmental conditions were maintained at 22°C ± 1°C, with a 12-hour dark/light cycle. Although movement was restricted for mice in the HLU group, they had unrestricted access to standard rodent chow and water. The experiment ended with isoflurane (Ante Animal Husbandry Technology Co. LTD, China) anesthesia (2% isoflurane,inhaled until complete anesthesia) followed by euthanasia (10L/min CO2 for 10min). The study protocol was approved by the Institutional Animal Care and Use Committee (IACUC) of Chongqing Medical University, with the approval number IACUC-CQMU-2024-03170.

### 2.2 MicroCT analysis

The right femurs were isolated from each mouse, and after meticulous removal of soft tissues, the femurs were fixed overnight in 4% neutral buffered formaldehyde (Biosharp, China). Subsequent Micro-CT analysis was conducted at Xuanzun Bio. (Chongqing, China). The bones were scanned using a Bruker Micro-CT Skyscan 1276 system (Kontich, Belgium) with the following parameters: tube current 200uA, voltage 70KV, whole body scan, resolution of 6.534165μm, exposure time of 350ms, and a scan angle of 180 degrees. Calibration was performed using a phantom, which was provided and standardized by the equipment manufacturer and scanned under identical conditions. A total of 2301 slices were imaged and subsequently reconstructed using NRecon software (Version 1.7.4.2). The reconstructed images were analyzed with CTAn software (Version 1.20.3.0). Analysis commenced with the growth plate sections, with manual delineation for trabecular and cortical bone image segmentation in selected areas. The analysis provided several key parameters: trabecular bone mineral density (Tb.BMD, g/cm^3), trabecular bone volume fraction (BV/TV, %), trabecular thickness (Tb.Th, mm), number of trabeculae (Tb.N, 1/mm), structural model index (SMI), and cortical bone mineral density (Ct.BMD, g/cm^3) for six specimens (33).

### 2.3 Primary cell culture and gene expression quantification

After euthanasia, the femurs were extracted from the mice, with muscles meticulously removed. The bone marrow was then flushed out using sterile phosphate-buffered saline (PBS) (Biosharp, China). Subsequent to centrifugation at 200xg, the bone marrow was resuspended in α-MEM complete medium (Gibico, USA) supplemented with 10% FBS (Shanghai Sangon, China) and 1% double antibiotics (Solabio, China). The mixture was cultured at 37°C within a 5% CO2 atmosphere for three days to diminish the impact of hematopoietic stem cells (34, 35). Post culture, non-adherent cells were discarded, and total RNA was harvested using the AG RNAex Pro kit (Accurate Biology, China). This RNA was then reverse transcribed into cDNA using the Evo M-MLV reverse transcription kit (Accurate Biology, China) for subsequent analysis. Real-time quantitative polymerase chain reaction (qPCR) was performed utilizing the SYBR Green Pro Taq HS premixed qPCR kit (Accurate Biology, China) on the Gentier 96R real-time PCR system (Tianlong Science and Technology, Xi’an, China). Relative mRNA expression levels were standardized against the internal reference gene GAPDH employing the 2(-Delta Delta C(T)) method to assess expression alterations (36, 37). The primers used are listed in **Supplementary Table 1**.

### 2.4 Histological analysis

After euthanasia, all non-bony tissues were removed from the right femur as thoroughly as possible. The femur was then fixed overnight in 4% paraformaldehyde (Biosharp, China) and subsequently decalcified for 14 days in sterile 14% disodium ethylenediaminetetraacetic acid (EDTA-2Na) at pH 7.4. Following decalcification, the femur was embedded in paraffin and sectioned into 5 μm slices using longitudinal coronal cuts. The sections were stained with hematoxylin and eosin (H&E, Servicebio, China) for general histology. Osteoclasts were identified using TRAP staining (38) (TRAP staining kit, Servicebio, China). The development of growth plate cartilage was assessed using Alcian Blue/Periodic Acid-Schiff (AB-PAS) staining (39) (AB-PAS staining solution, Servicebio, China).

### 2.5 Data source

Osteoporosis-related transcriptome datasets GSE100930 and GSE80614, along with single-cell dataset GSE147287, were downloaded from Gene Expression Omnibus database (https://www.ncbi.nlm.nih.gov/gds). The GSE100930 dataset, consisting of RNA sequencing data from three flight cell samples and three ground cell samples cultured in osteogenic medium, which cell samples were primarily human bone marrow MSCs (hBMSCs) (24). GSE80614 dataset, consisting of RNA sequencing data from 66 human mesenchymal stromal cell (hMSC) samples at three different phases of differentiation [phase I (0 h, 0.5 h, 1 h, 2 h and 3 h), phase II (6 h, 12 h and 24 h), phase III (48 h, 72 h and 96 h)] in osteogenic and adipogenic differentiation medium (25), and GSE147287 dataset, consisting of single-cell RNA sequencing data from freshly isolated CD271+ BM-derived mononuclear cells (BM-MNCs) of osteoporosis patients (26) were included in this study. Furthermore, 203 focal adhesion-related genes (FARGs) were obtained from Kyoto encyclopedia of genes and genomes (KEGG) database (https://www.genome.jp/kegg/) (**Supplementary Table 2**).

### 2.6 Identification and enrichment analysis of DE-FARGs

Differentially expressed genes (DEGs) between cases and control samples were obtained in the GSE100930 dataset by limma software package (v 3.56.2) (40),in which an empirical Bayesian approach was applied to adjust the t - statistic and the p - value using the eBayes function on the basis of lmFit to improve the accuracy and stability of the differential expression analysis. The information of differentially expressed genes was extracted using the topTable function, and |log2FC| ≥ 1 and p.value < 0.05 were set as the criteria to screen for significant differentially expressed genes. Then, volcano plot and heat map of DEGs were plotted using ggplot2 package(v 3.3.6) (41) and Heatmap3 package (v 1.1.9) (42), respectively. Then, the genes associated with FA were acquired by overlapping DEGs and FARGs, defined as DE-FARGs. To understanding the potential biological functions and signaling pathways associated with DE-FARGs, Gene ontology (GO) and KEGG enrichment analysis of DE-FARGs were completed *via* ClusterProfiler package (v 4.8.2) (43) (adj.P < 0.05). Among them, GO enrichment analysis was performed using the enrichGO function and KEGG enrichment analysis was performed using the enrichKEGG function. On this basis, we simultaneously performed KEGG analysis on 203 FARGs to compare with the DE-FARGs enrichment results.

### 2.7 Identification and enrichment analysis of FA-related candidate genes in osteogenesis and adipogenesis

In order to preprocess and transform the transcriptome data from GSE80614 dataset, so as to improve the accuracy and reliability of the subsequent soft clustering analysis, Gene Set Variation Analysis (GSVA) was executed among the three distinct differentiation phases in 66 hMSC samples undergoing OS (n = 33) and AD (n = 33) differentiation (phase I vs phase II, phase I vs phase III, and phase II vs phase III) by GSVA package (v 1.48.3) (44) (P < 0.05). Databases such as Molecular Signatures Database (MSigDB) were used as background gene sets in this process. Then, soft clustering analyses were conducted on OS and AD samples at 11 distinct differentiation time points (0 h, 0.5 h, 1 h, 2 h, 3 h, 6 h, 48 h, 72 h and 96 h) across three phases from GSE80614 dataset using Mfuzz package (v 2.62.0) (45) to explore the genes that contribute to sustained up-regulation or sustained down-regulation of hMSC during various differentiation time points in OS and AD.

In order to further identify the OS and AD genes related to FA in the development of osteoporosis, we intersected the previously identified DE-FARGs and those obtained genes through soft clustering for OS and AD processes. The overlapping genes were defined as FA-related candidate genes in OS and AD. Subsequently, to deepen reveal the potential biological functions and underlying mechanisms, GO and KEGG enrichment analyses were conducted separately for the FA-related candidate genes in OS and AD using ClusterProfiler package (adj.P < 0.05). Similarly, the enrichGO function was used for GO enrichment analysis, and the enrichKEGG function was used for KEGG enrichment analysis.

### 2.8 Identification and analysis of FA-related key gene in OS and AD

For further exploration of FA-related key gene in OS and AD, the FA-related candidate genes in OS and AD were initially uploaded to the online database search tool for the retrieval of interacting genes (STRING) (https://cn.string-db.org/) for protein-protein interaction (PPI) network construction (confidence value > 0.4). Afterwards, the Degree algorithm of the cytoHubba tool was used to filter the top 5 scores as FA-related key gene in OS and AD. The visualization of this network was achieved with Cytoscape software (v 3.9.1) (46).

Moreover, with the intention of understanding the mechanism of key genes, mRNALocater database (http://bio-bigdata.cn/mRNALocater/) was utilized to predict subcellular localization of key genes. And the chromosomal distribution of key genes was visualized using RCircos package (v 1.2.2) (47). In addition, the expression levels of key genes in OS (n = 33) and AD samples (n = 33) from GSE80614 dataset were analyzed.

### 2.9 Gene set enrichment analysis of key genes

In order to gain insights into biological functions and pathways associated with key genes, GSEA of each key gene was executed by ClusterProfiler package (adj.P < 0.05) *via* the correlation from the expression levels of key genes and other genes. The c2: KEGG was acquired from the msigdbr package (v 7.5.1) as background gene set.

### 2.10 Construction of regulatory network

Efforts were made identify other genes connected with key genes function, and a Gene-Gene Interaction (GGI) network was constructed through the use of the GeneMANIA database (http://genemania.org), using the key genes as input. Besides, to further predict correlation drugs for key genes, we utilized the Comparative Toxicogenomics Database (CTD) database (https://ctdbase.org/) to identify drugs associated with the key genes (Reference literature > 2), and the results were visualized using Cytoscape software.

### 2.11 Single-cell analysis

The comprehensive analysis of single-cell sequencing data was analyzed by Seurat package (v 5.0.0) (48). First, in order to exclude low-quality data arising from cell damage or failures in library preparation, we performed quality control on single-cell sequencing data based on the following criteria: (a) cells with gene counts greater than 200, and greater than three cell-covered were retained, (b) cells with gene counts less than 2500 and gene expression count less than 6000 were retained, (c) cells with less than 10% of mitochondrial gene expression were retained. Then, FindVariableFeatures function combined with VST to screen the top 2000 highly variable genes. For further evaluation and analysis of cell cluster, linear dimensionality reduction was performed *via* gene expression in each cell. Statistically significant principal components (PCs) were identified by means of the JackStrawPlot function based on the null-distribution permutation test, and dimensionality reduction was performed using principal component analysis (PCA).Subsequently, unsupervised cluster analysis of the cell after dimensionality reduction was done using FindNeighbors and FindClusters functions (resolution = 0.6), followed by t-distributed stochastic neighborhood embedding (t-SNE) was employed to visualize the cell clusters. Finally, we annotated the cell clusters based on the marker genes in the literatures (26). Cells with high expression in key genes were selected as key cells associated with OS and AD.

### 2.12 GSEA of key cells

With the aim of investigating potential biological roles and signaling pathways of key cells associated with OS and AD, in the first place, DEseq2 package (v 1.42.0) (49) was used to determine and rank according to fold change in differential expression of a specific key cell cluster relative to other key cell clusters within GSE147287 dataset. The specific parameters were set as follows: ‘min - genes’ was set to 15, ‘max - genes’ was set to 500. The ‘p - value’ threshold was set to 0.05, ‘FDR’ threshold was set to 0.25. Next, based on rank results, GSEA was executed through ClusterProfiler package (P < 0.05).

### 2.13 Analysis of cell clusters communication

Cell communication analysis was conducted to examine the expression and pairing of receptors and ligands in cell clusters, aiming to infer interactions between different cells. The CellChat package (v 1.6.1) (50) was employed to screen out receptor-ligand pairs present in GSE147287 dataset, where the probability threshold was set to 0.05. Then, leveraging intercellular receptor ligands to construct comprehensive cell-cell communication networks.

### 2.14 Cell cycle phase and pseudo-time analysis of key cells

For the purpose of comprehending the phases that cells undergo, thus enhancing our comprehension of cell behavior and interactions, cell cycle annotation was conducted by utilizing the G2/M and S phase marker genes provided within Seurat package. To this end, our study aimed to investigate the differentiation trajectory and evolution of key cells during development, we utilized monocle2 package (v 2.30.0) (51) for conducting pseudo-time series analysis, focusing on the gene expression changes over time in the key cells.

## 3 Results

### 3.1 Hindlimb unloading in mice: reduced bone density, altered gene expression in bone marrow mesenchyml stem cells, and bone microstructure disruption

**Figure 1:**
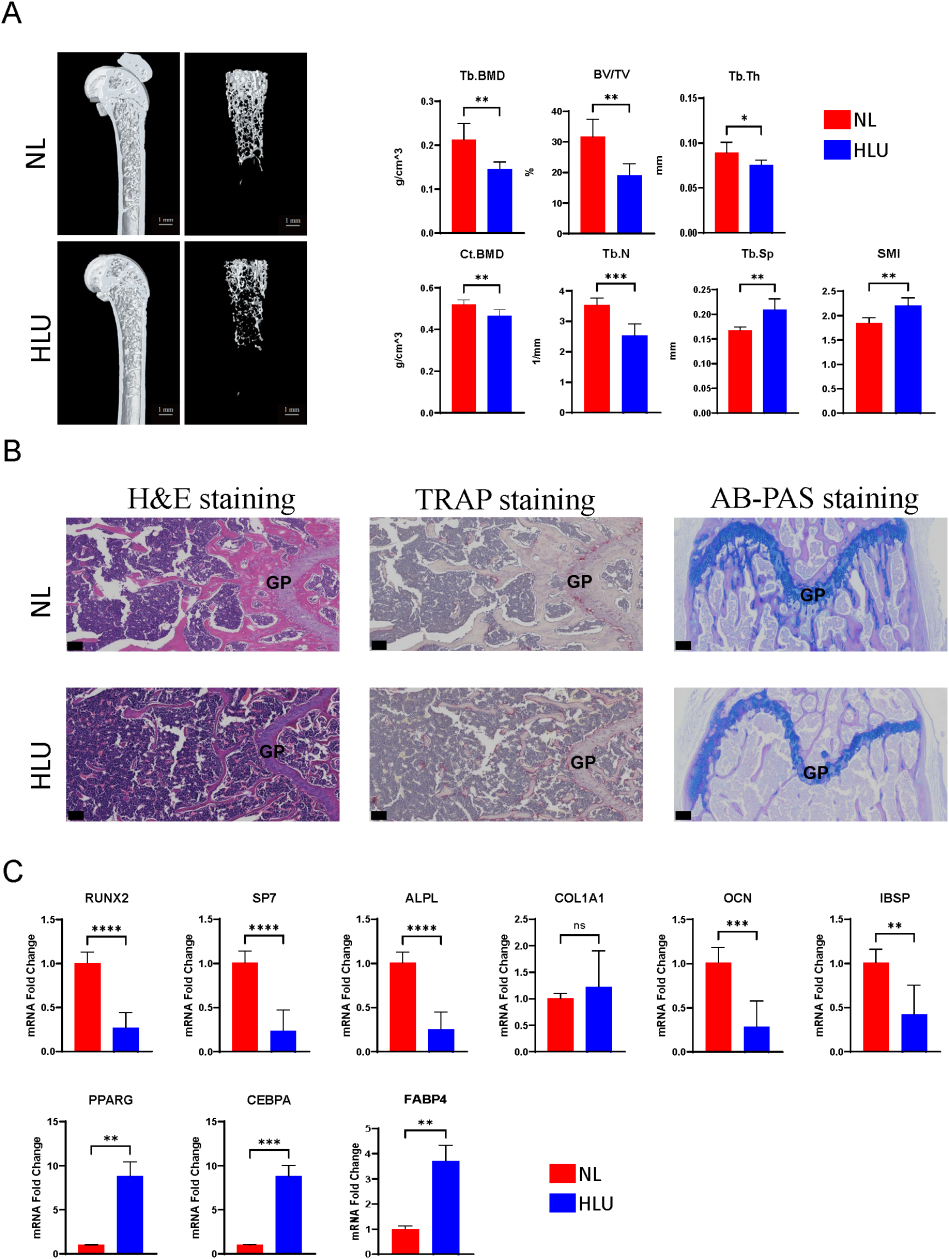
Micro-CT analysis, histological staining, and gene expression studies. **(A)** Micro-CT analysis of the HLU/Tail-suspension model (n=6, t-test, *p <0.05, **p <0.01, ***p < 0.001, ****p < 0.0001). **(B)** Histological analyses, including hematoxylin and eosin staining, TRAP staining, AB-PAS staining. scale bar: 50μm **(C)** The expressions of Osteogenic/Adipogenic genes in BMSCs (n=3, t-test, ns=no significance, *p <0.05, **p <0.01, ***p < 0.001, ****p < 0.0001).

Micro-CT analysis of the HLU/Tail-suspension model demonstrated significant bone density changes after two weeks of hindlimb unloading had been implemented. The trabecular bone density (Tb. BMD) in mice was markedly reduced to 0.144531 g/cm^3—a decline of over 31.7% compared to the control group, which maintained a normal hindlimb loading and exhibited a Tb. BMD of 0.211837 g/cm^3. Similarly, we observed changes in cortical bone parameters. The difference in bone density (Ct. BMD) exceeded 10%, and the proportion of cortical bone area (Ct. Ar) to total tissue area (Tt. Ar) also showed a corresponding difference, with the control group at 41.2717% and the HLU group at 38.665% (**Supplementary Table 4**). Additional notable differences included a decrease in the bone volume fraction (BV/TV) to 31.7% in the unloading group, compared to 19.2% in the control group. The analysis also revealed a deterioration in the microstructure of bone tissue, as evidenced by changes in the average number of trabeculae (Tb. N) and the average thickness of trabeculae (Tb. Th) (n=6, p<0.01, t-test) (**Figure 1A**). Cells extracted from mouse bones marrow were cultured for three days at 37°C, 5% CO2, and then analyzed by qPCR, which showed significant differences in the expression of osteogenesis-related genes. Among the six osteogenesis-related genes commonly used to assess osteogenic differentiation of stem cells or osteoblasts, the expression of all genes, except for COL1A1, differed significantly between groups. Specifically, the expression levels in bone marrow stromal cells of the hindlimb unloading group were significantly lower than those in the normal weight-bearing group. (n=3, p<0.01, t-test) (**Figure 1C**). Contrarily, genes associated with adipogenic differentiation, including CEBPA, FABP4, and PPARG, were upregulated in the mesenchymal stem cells of the hindlimb unloading group, suggesting a shift from osteogenic to adipogenic differentiation (**Figure 1C**). Histological analyses corroborated these findings. Hematoxylin and eosin staining indicated structural damage to the bone, with an increase in round or oval vacuoles in the HLU group, signifying an accumulation of fat granules within the marrow cavity (**Figure 1B**). TRAP staining revealed heightened osteoclast development in the HLU group (**Figure 1B**). AB-PAS staining indicated restricted differentiation of growth plate chondrocytes—primary spongy bone derived from growth plate chondrocytes was markedly atrophied, and the accumulation of glycosaminoglycans (blue staining in AB-PAS staining) in the primary spongy bone was significantly lower in the HLU group compared to the normal loading group (**Figure 1B**).

### 3.2 A sum of 35 DE-FARGs were acquired

**Figure 2:**
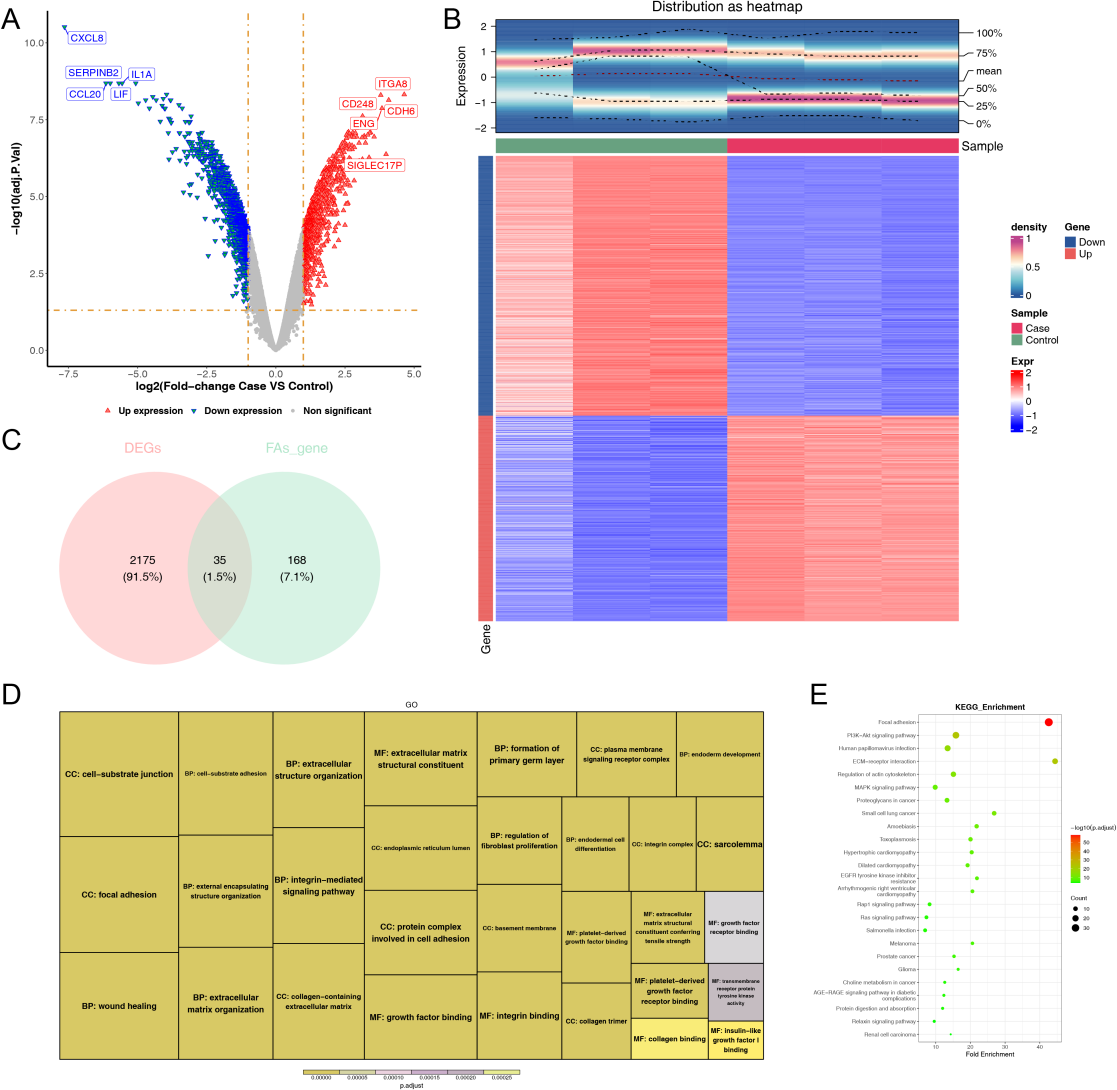
Comprehensive analysis of differentially expressed genes: volcano map, density heatmap, intersection with FARGs, and functional enrichment. **(A)** Volcano map of differentially expressed genes. Significantly up-regulated genes in red and significantly down-regulated genes in blue. **(B)** Density heatmap of differentially expressed genes. The upper section displayed the heatmap of gene distribution, while the lower section presented the heatmap of gene expression. The horizontal axis represented the samples, and the vertical axis represented the differentially expressed genes. The colors indicated the normalized gene expression levels, with red representing high expression and blue representing low expression. The term “sample” denoted the grouping of the samples. **(C)** Venn diagram of the intersection of DEGs and FARGs. **(D, E)** GO and KEGG enrichment analysis of DE-FARGs. The size of the boxes represented the number of genes included, while the color indicated the significance.

A sum of 2,210 DEGs, including 976 up-regulated and 1,234 down-regulated were screened out between case and control samples from the GSE100930 (**Figures 2A, B**). By overlapping these DEGs with FARGs, we acquired 35 DE-FARGs (**Figure 2C**). The biological activities and pathways of 35 DE-FARGs were further investigated by GO and KEGG enrichment analysis, as demonstrated in **Figures 2D, E**. The results indicated that DE-FARGs were significantly associated with 889 GO items [759 biological process (BP), 70 cellular components (CC) and 60 molecular functions (MF)] (adj.P < 0.05), such as “focal adhesion”, “cell−substrate adhesion”, “nplatelet-derived growth factor binding”. Meanwhile, under the KEGG analysis, 72 pathways were enriched (adj.P < 0.05), like “focal adhesion”, “glutathione metabolism”, “PI3K-Akt signaling pathway”, “ECM-receptor interaction”. This result indicated that the enrichment of FARGs was relatively high within the overall DEGs. This finding suggested that focal adhesion-related biological functions and signaling pathways were involved in the occurrence and development of osteoporosis. In addition, the results of KEGG analyses of 203 FARGs were compared with the results of DE-FARGs enrichment. The top ten ranked pathways were found to be more similar between the two (**Supplementary Table 3**).

### 3.3 Totally 14 FA-related candidate genes in OS and 13 FA-related candidate genes in AD were identified

**Figure 3:**
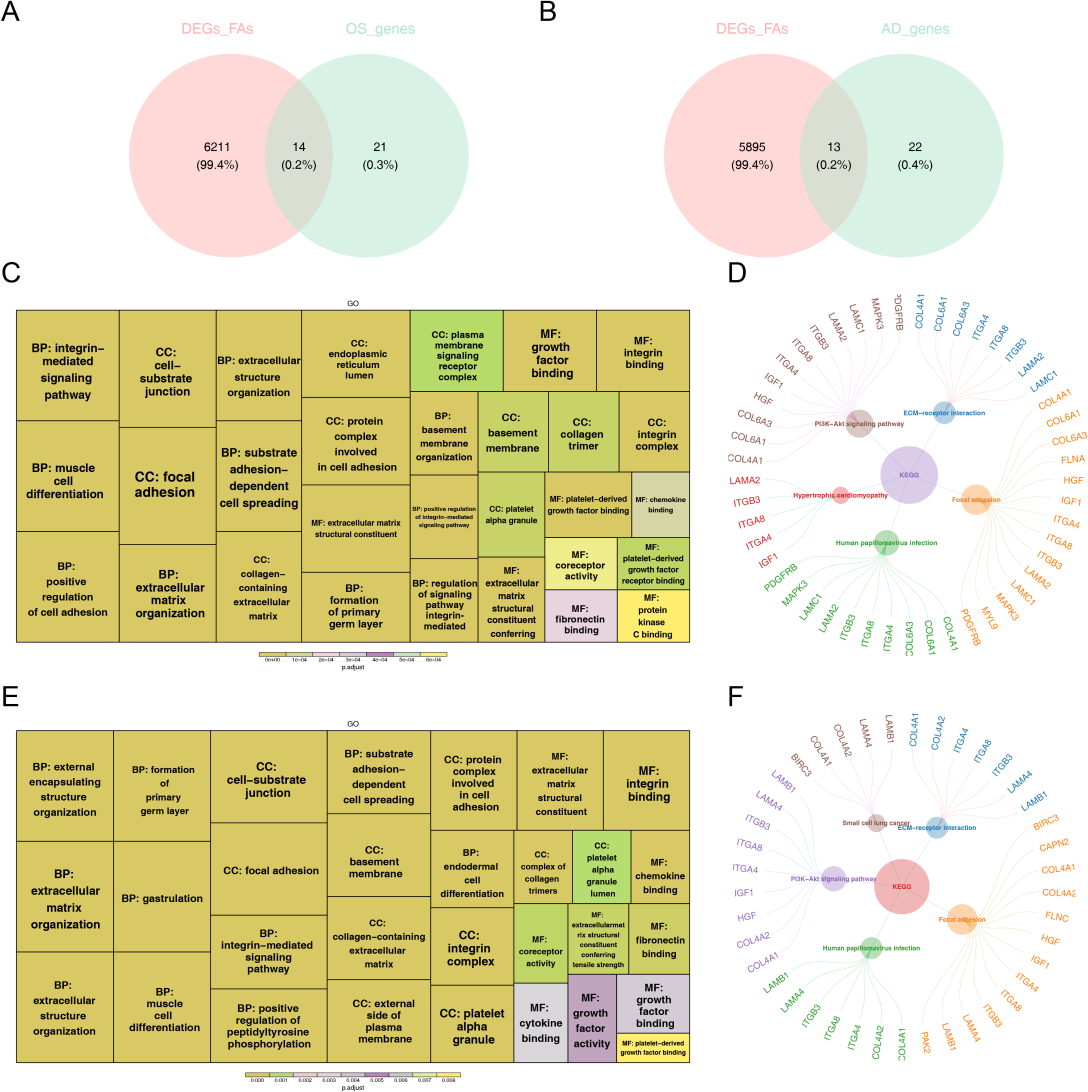
The functional enrichment analysis of osteogenesis and adipogenesis-related genes intersecting with DE-FARGs. **(A)** Venn diagram of the intersection of DE-FARGs with osteogenic genes obtained by soft clustering. **(B)** Venn diagram of the intersection of DE-FARGs with adipogenic genes obtained by soft clustering. **(C)** GO enrichment analysis of osteogenic candidate genes. The size of the boxes represented the number of genes included, while the color indicated the significance. **(D)** KEGG enrichment analysis of osteogenic candidate genes. **(E)** GO enrichment analysis of adipogenic candidate genes. The size of the boxes represented the number of genes included, while the color indicated the significance. **(F)** KEGG enrichment analysis of adipogenic candidate genes.

We utilized GSVA to evaluate changes in pathway enrichment among 66 hMSC samples undergoing OS (n = 33) and AD (n = 33) differentiation in GSE80614 dataset, and the results were shown in **Supplementary Figure 1**. Thereafter, soft clustering analyses were conducted to further screen out FA-related candidate genes in OS and AD. Explicitly, we selected Cluster 5 with sustained up-regulation and Cluster 10 with sustained down-regulation in OS samples, as well as Cluster 7 with sustained up-regulation and Cluster 6 with sustained down-regulation in AD samples, for subsequent analysis (**Supplementary Figures 2A, B**). By intersecting the 35 DE-FARGs with Cluster 5 and Cluster 10 in OS samples, as well as Cluster 6 and Cluster 7 in AD samples, a total of 14 FA-related candidate genes in OS and 13 FA-related candidate genes in AD were identified (**Figures 3A, B**).

Next, both 14 FA-related candidate genes in OS and 13 FA-related candidate genes in AD were incorporated into GO and KEGG analysis to further explore some of relevant signaling pathways and potential biological mechanisms underlying the presence of candidate genes. Concretely, FA-related candidate genes in OS and AD were primarily co-associated with “focal adhesion”, “substrate adhesion-dependent cell spreading”, “protein complex involved in cell adhesion”, “extracellular matrix structural constituent”, and “muscle cell differentiation”, etc., in GO entries (**Figures 3C, D**), and were mainly co-enriched in “PI3K-Akt signaling pathway”, “ECM-receptor interaction”, “human papillomavirus infection”, and “focal adhesion” and other KEGG pathways (**Figures 3E, F**). The findings further establish the pivotal role of focal adhesion-related biological functions and signaling pathways in the pathogenesis of osteoporosis.

### 3.4 A total of five FA-related key genes in OS and five FA-related key genes in AD were identified

**Figure 4:**
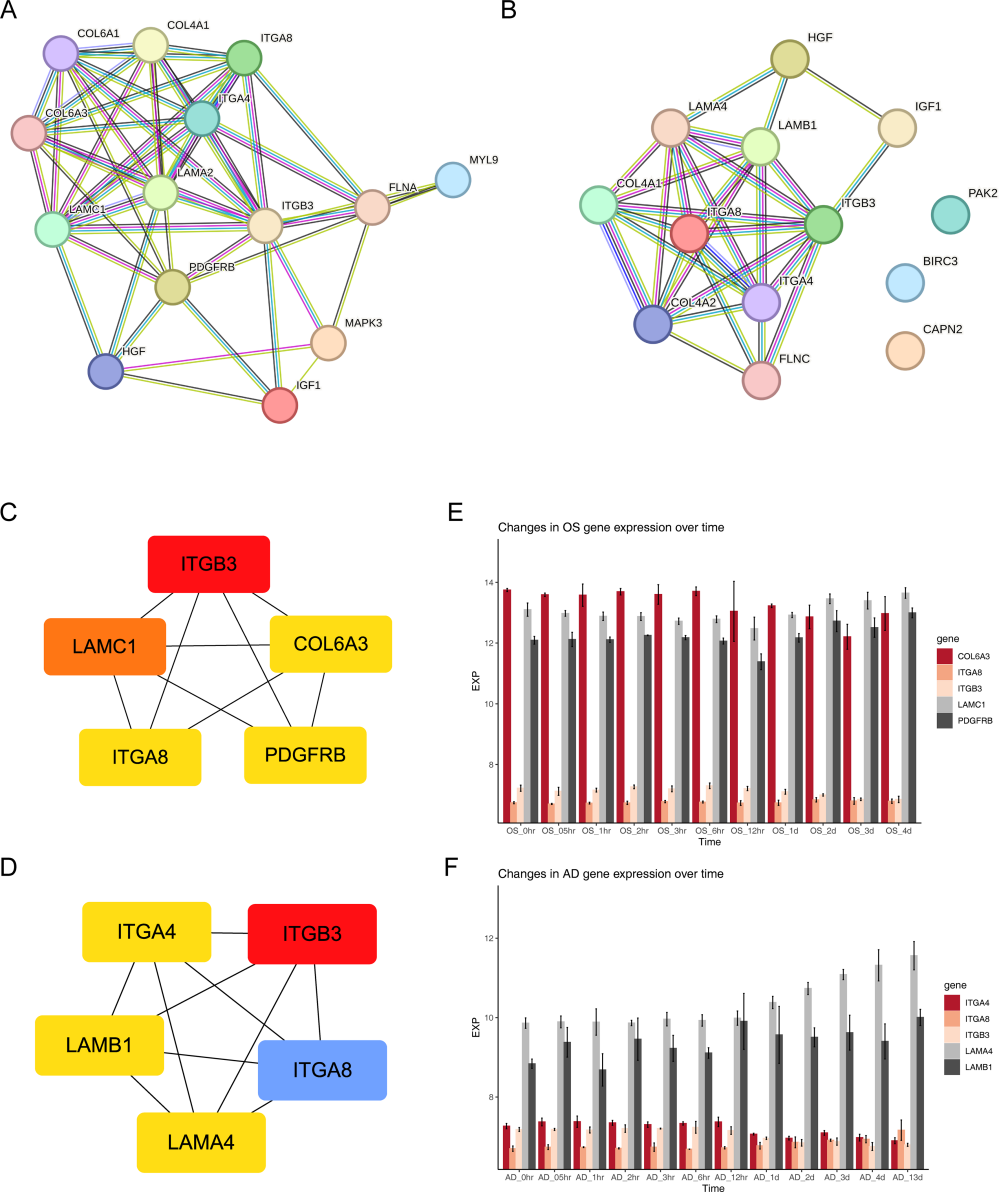
Exploring protein interactions and gene expression patterns in osteogenesis, adipogenesis, osteoarthritis, and Alzheimer’s disease through PPI networks and time-course analysis. **(A)** PPI network of 14 FA-related candidate genes in osteoarthritis. **(B)** PPI network of 13 FA-related candidate genes in Alzheimer’s disease. **(C)** PPI network of key osteogenic proteins. **(D)** PPI network of key adipogenic proteins. **(E)** Expression patterns of key osteogenic genes at 11 different time points of osteogenic differentiation in the GSE80614 dataset. **(F)** Expression patterns of key adipogenic genes at 11 different time points of adipogenic differentiation in the GSE80614 dataset.

Two protein-protein interaction (PPI) networks were constructed based on the 14 FA-related candidate genes in osteoarthritis (OS) and the 13 FA-related candidate genes in AD to identify key genes (**Figures 4A, B**). From this analysis, we identified five FA-related key genes in OS (ITGB3, LAMC1, COL6A3, ITGA8, and PDGFRB) (**Figure 4C**) and five FA-related key genes in AD (ITGB3, ITGA4, LAMB1, ITGA8, and LAMA4) (**Figure 4D**). Notably, ITGB3 and ITGA8 were common key genes in both conditions. Compared to the normal load (NL) group, significant differences were observed in the mRNA expression of ITGB3, LAMC1, COL6A3, ITGA8, PDGFRB, ITGA4, LAMB1, and LAMA4 genes in the disuse osteoporosis animal model. Apart from ITGB3, LAMA4, LAMB1, and COL6A3, all other key genes were upregulated. consistent with the results from the GSE100930 dataset (**Table 1**). In the GSE100930 dataset, the full gene expression was as follows: the AD group showed down-regulation of IGF1, BIRC3, and up-regulation of PAK2, LAMA4, ITGB3, ITGA8, LAMB1, ITGA4, FLNC, HGF, COL4A1, COL4A2. the OS group showed IGF1, down-regulation of MYL9, LAMA2 ITGA8, COL6A3, COL6A1, COL4A1, HGF, LAMC1, ITGA4, MAPK3, ITGB3, FLNA upregulated.

**Table 1:** Differences in the expression of key genes within the GSE100930 dataset; Soft clustering results for key genes within the GSE80614 dataset; qPCR validation of key genes.

GENE	LogFC in GSE100930	Soft Cluster in Supplementary Figure 2	qPCR Fold Change in HLU model	Comparison of directions of change
ITGA4	1.131646667	AD Cluster 6	1.630217918593750	HLU: ↑, GSE: ↑
ITGA8	4.642286667	OS Cluster 5; AD Cluster7	1.33225082352044	HLU: ↑, GSE: ↑
ITGB3	1.708796667	OS cluster 10; AD Cluster 6	0.207126740719072	HLU: ↓, GSE: ↑
LAMA4	1.236523333	AD Cluster 7	0.149554312065873	HLU: ↓, GSE: ↑
LAMB1	2.663303333	AD Cluster 7	0.886811840237692	HLU: ↓, GSE: ↑
LAMC1	1.80057	OS Cluster 5	1.603411972419880	HLU: ↑, GSE: ↑
COL6A3	2.188483333	OS Cluster 10	0.641643171945876	HLU: ↓, GSE: ↑
PDGFRB	2.406236667	OS Cluster 5	1.723945470367960	HLU: ↑, GSE: ↑

↓ indicates downregulation of gene expression, and ↑ indicates upregulation of gene expression. In the HLU model, this is relative to the control group, while in GSE, LogFC > 1 indicates gene upregulation.

Subsequently, we predicted the subcellular localization of key genes using mRNALocater database (**Supplementary Figures 3A, B**). Evidently, ITGB3, COL6A3, ITGA8, and ITGA4 were primarily distributed in nucleus and cytoplasm. LAMC1 and LAMB1 were mainly localized in the nucleus. PDGFRB was predominantly found in the cytoplasm and endoplasmic reticulum, while LAMA4 exhibited main distribution in the nucleus, cytoplasm, and extracellular region. Chromosome locations were identified as follows: ITGB3 on chromosome 17, LAMC1 on chromosome 1, COL6A3 and ITGA4 on chromosome 2, ITGA8 on chromosome 10, PDGFRB on chromosome 5, LAMB1 on chromosome 7, and LAMA4 on chromosome 6 (**Supplementary Figures 3C, D**). In addition, the expression levels of key gene in different phases of differentiation from GSE80614 dataset were analyzed. Interestingly, we observed higher expression of COL6A3, LAMC1, and PDGFRB in OS samples, whereas LAMA4 and LAMB1 showed elevated expression in AD samples (**Figures 4E, F**).

### 3.5 FA-related key genes in OS and AD were enriched in genetic information processing-related and cellular processes-related signaling pathways

**Figure 5:**
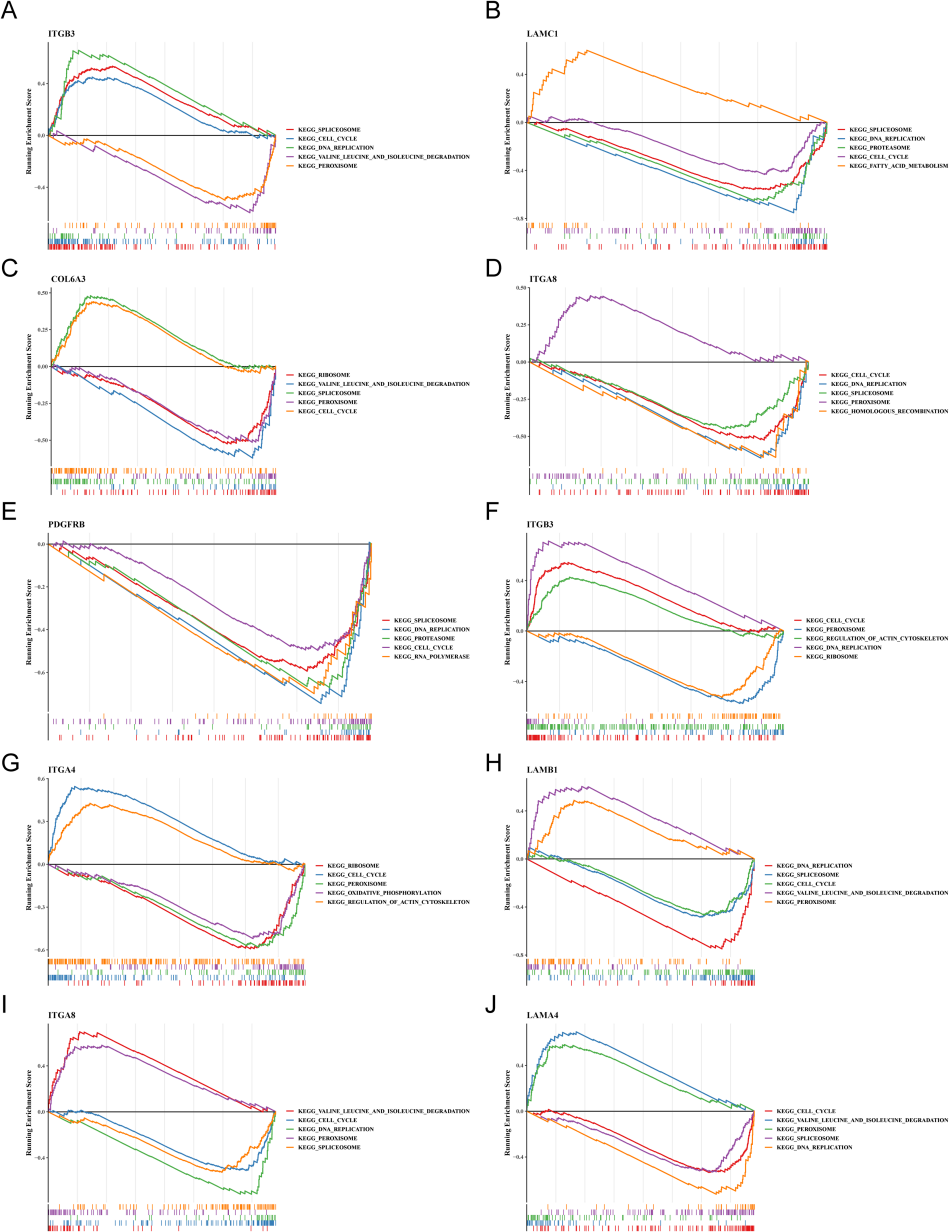
The trends in GSEA enrichment of key osteogenic and adipogenic genes. **(A–E)** Trend of GSEA enrichment of key osteogenic genes. **(F–J)** Trend of GSEA enrichment of key adipogenic genes.

Likewise, to understand the functions and potential pathways involved in key genes, GSEA were executed. Notably, in OS samples, ITGB3, LAMC1, COL6A3, ITGA8 and PDGFRB were co-enriched in “cell cycle”, ITGB3, LAMC1, ITGA8 and PDGFRB were co-enriched in “DNA replication”. This indicated that these genes might have regulated the proliferation and differentiation of osteoblasts by influencing cell cycle processes and DNA replication. ITGB3, LAMC1, COL6A3 and ITGA8 were co-enriched in “spliceosome”. This suggested that they might have been involved in the post-transcriptional regulation of osteogenesis-related genes. ITGB3, COL6A3 and ITGA8 were co-enriched in “peroxisome”. It was inferred that peroxisome function might have been involved in mediating osteoblasts. (**Figures 5A–E**). Similarly, in AD samples, ITGB3, ITGA4, LAMB1, ITGA8 and LAMA4 showed co-enrichment in “cell cycle” and “peroxisome”, ITGB3, LAMB1, ITGA8 and LAMA4 were co-enriched in “DNA replication”, LAMB1, ITGA8 and LAMA4 were co-enriched in “spliceosome” and “valine leucine and isoleucine degradation” (**Figures 5F–J**). These findings revealed that FA-related key genes in OS and AD might influence the development of osteoporosis mainly through genetic information processing and cellular processes.

### 3.6 Potential regulatory mechanisms and potential drugs of key genes

**Figure 6:**
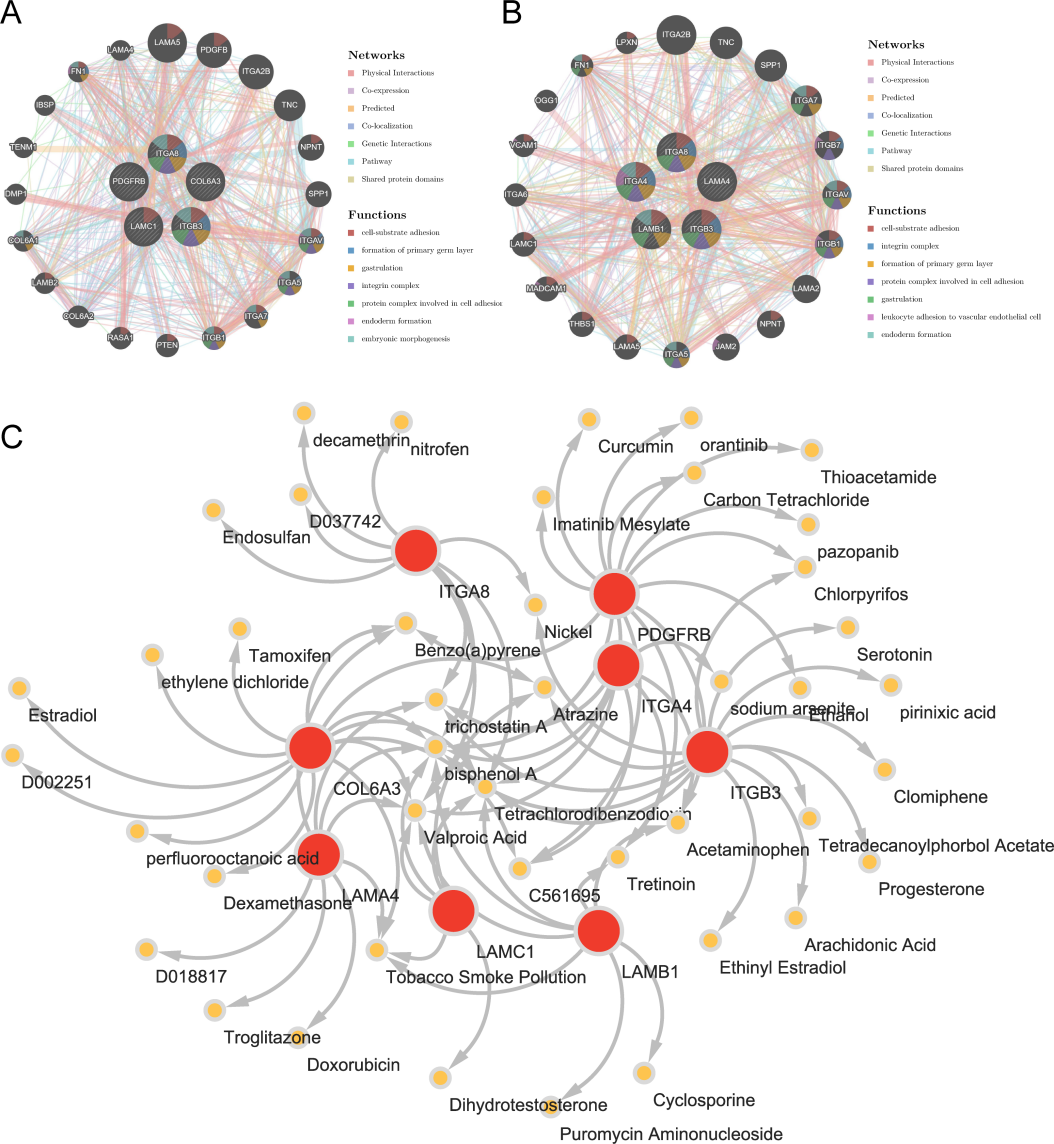
Insights from gene-gene interaction and drug association maps. **(A)** GGI network of key osteogenic genes. **(B)** GGI network of key adipogenic genes. **(C)** A network of 8 osteogenic-adipogenic related genes with 44 drugs.

Through analysis of the GGI network, we selected top 20 reciprocal genes associated with the functionality of key genes and determined their primary functions. The result indicated that FA-related key genes in OS were primarily involved in “cell-substrate adhesion”, “formation of primary germ layer”, and “gastrulation” (**Figure 6A**), whereas FA-related key genes in AS were mainly associated with “cell-substrate adhesion”, “integrin complex”, and “formation of primary germ layer” (**Figure 6B**). These functional differences reflected the diverse roles of focal adhesion (FA) pathways under different disease states, particularly in terms of cell-substrate adhesion and the formation of the primary germ layer, which were common factors in both osteogenesis (OS) and adipogenesis (AS). This finding suggested that FA-related key genes might have had impacts on the occurrence and progression of the diseases through these two functions. Furthermore, we had identified a total of 44 relevant compounds that were relevant to eight key genes. Remarkably, valprocid acid, tetrachlorodibenzodioxin, bisphenol A, trichostain A were among the hub drugs for these key genes (**Figure 6C**).

### 3.7 Chondrocyte precursor, adipocyte precursor and osteoblast precursor were identified as key cells associated with OS and AD

**Figure 7:**
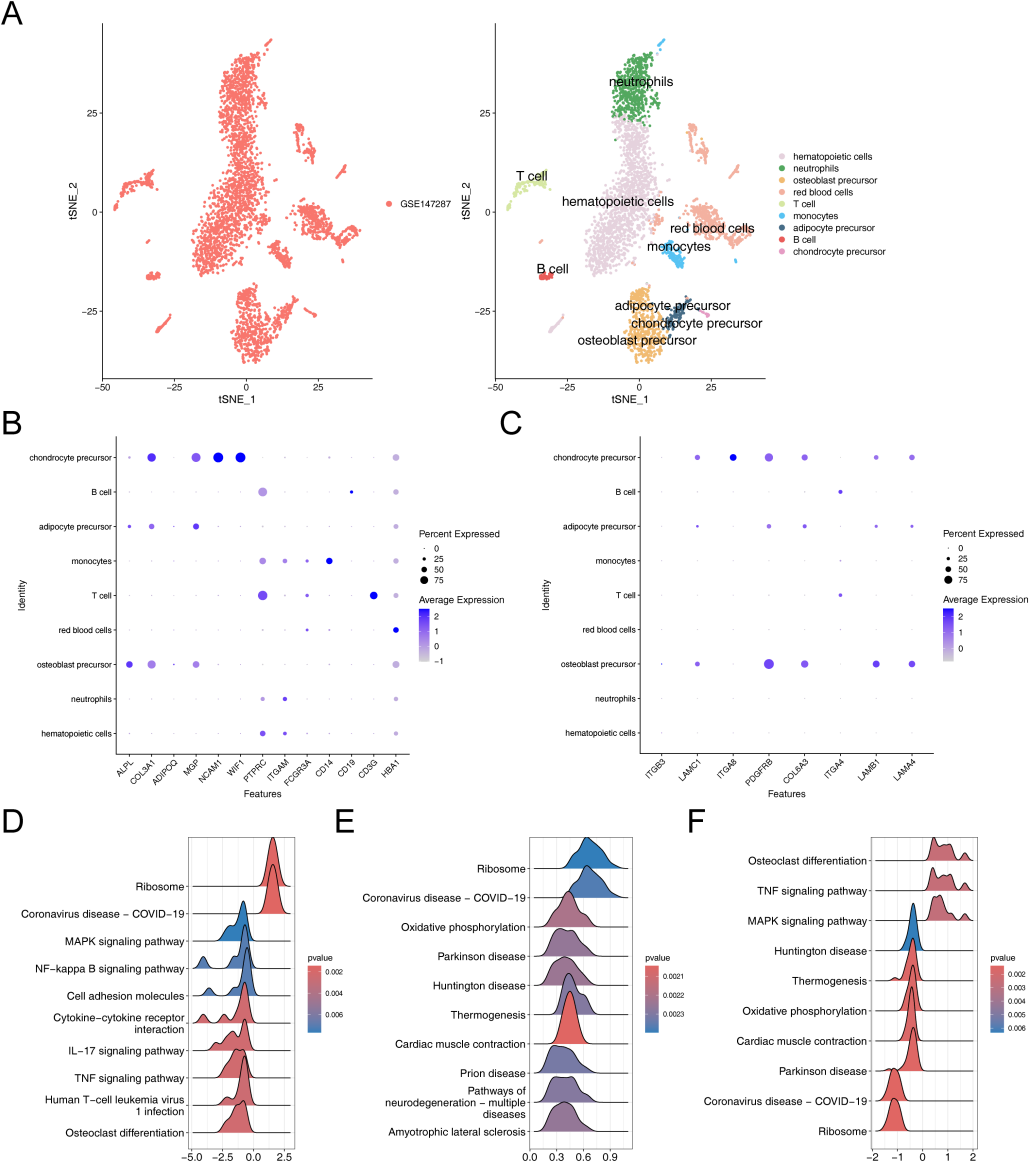
Comprehensive characterization of cellular heterogeneity and lineage-specific gene expression in osteogenic, adipogenic, and chondrogenic precursor cells. **(A)** TSNE clustering results after cell annotation. **(B)** Bubble chart of cell annotation. **(C)** Expression of key osteogenic and adipogenic genes in cellular taxa. **(D–F)** GSEA enrichment analysis of chondrogenic precursor cells, adipose precursor cells and osteogenic precursor cells.

The results before and after QC were showed in **Supplementary Figures 4A, B**. The cells that passed QC screening were used for subsequent analysis. After standard data processing, we selected 2,000 highly variable genes for study, which included the screening of genes with the most pronounced intercellular expression changes. The results obtained indicated the top 10 genes exhibiting the most pronounced intercellular expression changes were GNLY, IGHA1, IGKC, HBD, HBA1, IGHG4, HBA2, HBB, IGHG1, and IGLC2 (**Supplementary Figure 4C**). The PCA were conducted and the first 30 PCs were selected for subsequent analysis (**Supplementary Figures 4D, E**). Then, a sum of nine different cell clusters were annotated (**Figure 7A**), namely hematopoietic cells, neutrophils, osteoblast precursor, red blood cells, T cell, monocytes, adipocyte precursor, B cell, and chondrocyte precursor. The bubble plot demonstrated that the marker genes exhibited high specificity, thus the cells were named based on the marker genes (**Figure 7B**). Key genes exhibited high expression in chondrocyte precursor, adipocyte precursor and osteoblast precursor (**Figure 7C**). Since the differentiation of adipocytes and osteoblasts originated from the same precursor, it was ultimately determined that these three cell clusters as key cells associated with OS and AD.

The GSEA results suggested that chondrocyte precursor, adipocyte precursor and osteoblast precursor were co-enriched in pathways related to “ribosome”, “coronavirus disease-COVID-19”, chondrocyte precursor and osteoblast precursor showed co-enrichment in “MAPK signaling pathway”, “TNF signaling pathway” and “osteoclast differentiation”, adipocyte precursor and osteoblast precursor exhibited co-enrichment in “oxidative phosphorylation”, “parkinson disease”, “huntington disease”, “thermogenesis”, and “cardiac muscle contraction” (**Figures 7D–F**). These analyses revealed that these key cells were not only associated with environmental information processing-related signaling pathways but also closely related to genetic information processing-related signaling pathways, thereby enriching our comprehensive understanding of osteoporosis in relation to hMSC.

### 3.8 Cell communication, cell cycle phase, and pseudo-time trajectory inference

**Figure 8:**
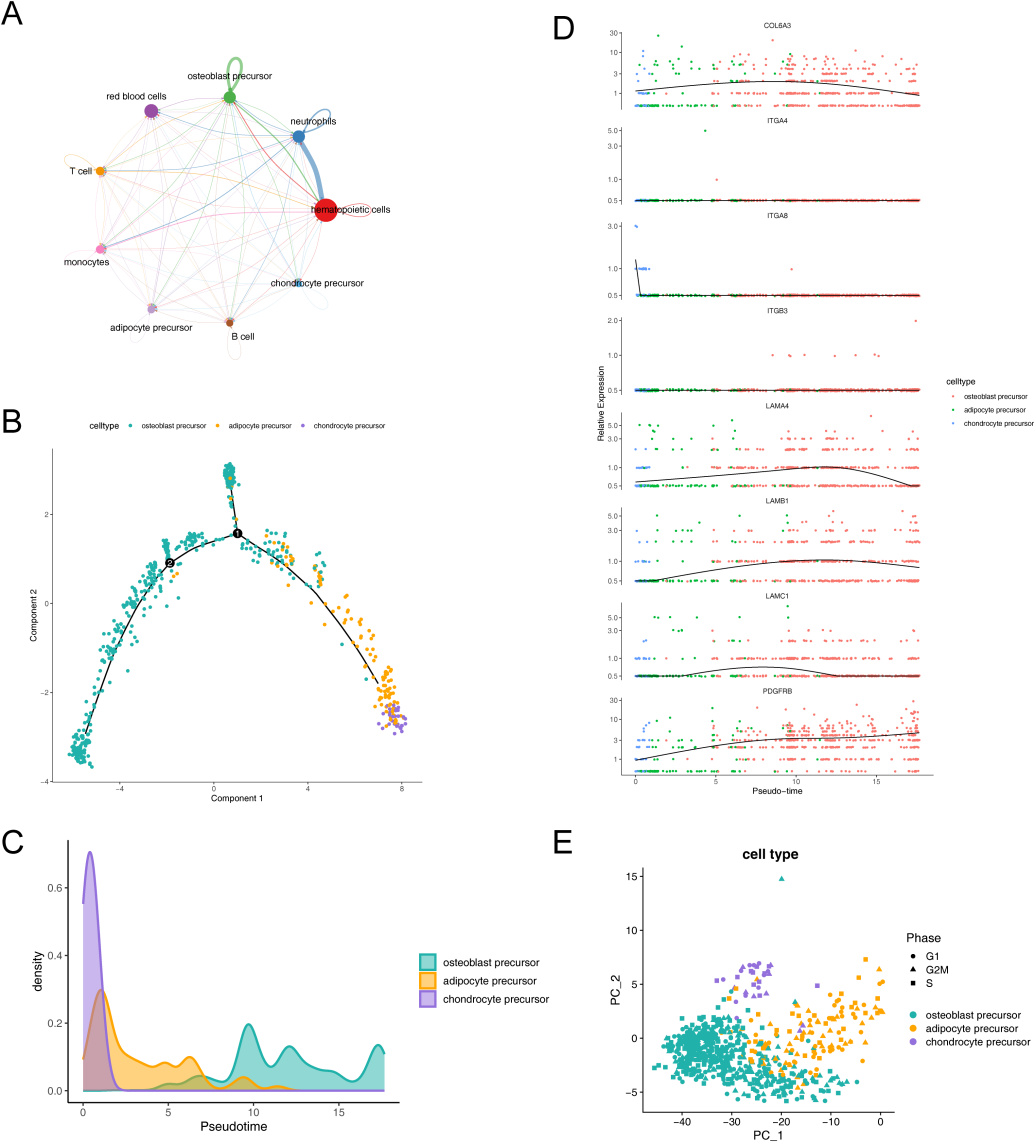
Dynamic interactions and differentiation trajectories of osteogenic-adipogenic associated cells and their lineage-specific precursors. **(A)** Diagram of communication between osteogenic-adipogenic associated cells and other cells. **(B)** Proposed time-series analysis of differentiation trajectories of osteogenic-adipogenic associated cells. **(C)** Differentiation processes of chondrogenic precursor cells, adipose precursor cells and osteogenic precursor cells. **(D)** Changes in the expression levels of eight key genes during cell differentiation. **(E)** Cell cycle PCA plots of chondrogenic precursor cells, adipose precursor cells and osteogenic precursor cells.

To investigate the expression and pairing of receptors and ligands in cell clusters, as well as to further elucidate the extent and pathways of intercellular interactions between different cell clusters within osteoporosis, we conducted an analysis of intercellular communication networks using CellChat (**Figure 8A**). Our observations revealed that hematopoietic cells exhibited a quantitative predominance within the overall sample. Specifically, we observed that neutrophils and hematopoietic cells displayed the strongest interactions. Among our identified key cells, we further observed varying degrees of interaction with other cell clusters, with osteoblast precursors exhibiting notably stronger interactions.

The trajectories of key cells were inferred by cell trajectory analysis and categorized into two parts (**Figure 8B**). It could be observed that chondrocyte progenitors were primarily distributed in the early developmental stage, while adipocyte progenitors were concentrated in the mid-developmental stage, and osteoblast progenitors mainly appeared in the late developmental stage. Additionally, branch point 1 was located at the early stage of the trajectory, where a large number of chondrocyte progenitors clustered near the right branch, while osteoblast progenitors were concentrated near the upper branch. Branch point 2 was situated at the mid-stage of the trajectory, but its associated biological functions and cell subpopulations remained to be further explored. **Figure 8C** illustrated the process of cell differentiation, where chondrocyte precursor rapidly confirmed their lineage at the early stage, followed by intermittent appearance of adipocyte precursor, and ultimately an extended duration in the osteoblast precursor differentiation zone. Further, we observed changes in the expression levels of eight key genes during cell lineage differentiation and simulated these changes by integrating the results of the proposed time series analysis with gene expression data. The genes LAMB1, LAMC1, COL6A3, and LAMA4 exhibited a pattern of initially increasing and then decreasing expression, while PDGFRB displayed a consistent increasing trend (**Figure 8D**).

Additionally, our analysis revealed a relatively even distribution of the identified key cells across the G1, G2M, and S phases, suggesting that the influence of cycle-related genes on our cell clustering was minimal (**Figure 8E**).

## 4 Discussion

Disuse osteoporosis is characterized by a rapid decline in bone mass and the degeneration of bone tissue microstructure. The weakening of osteogenic differentiation is manifested by a reduction in osteogenic marker genes and osteoblasts. Enhanced adipogenesis is reflected in an increase in adipogenic genes and adipocyte granules in the bone marrow (**Figure 1B** H&E staining). The rodent model (mouse HLU/Tail-Suspension model) was proposed by NASA at the end of the last century as an animal model scheme to simulate the adaptation syndrome shown by human femurs and tibias in space activities, where the bones experience reduced mechanical stress (27, 28, 52–55). Focal adhesion-related genes are a class of genes that couple two essential functions: influencing mesenchymal cell differentiation and mediating mechanotransduction. These genes encode components that establish physical connections between cells and the extracellular matrix. Mechanical signals from the extracellular environment—such as fluid flow, stress changes, vibration, or dynamic strain—are transmitted through these connections to the cytoskeleton, thereby enabling mechanosignal transduction from outside to inside the cell (56). Given that changes in the local stress environment are a direct cause of disuse osteopenia, and that signal transduction components encoded by focal adhesion-related genes can regulate the expression of osteogenic genes, such as alkaline phosphatase (57), it is also important to note that the microgravity environment has a significant impact on the biological behavior of bone marrow stromal cells (58). Therefore, exploring the potential functions of focal adhesion-related genes in microgravity-induced disuse osteoporosis is crucial, as it may provide novel insights into the mechanisms underlying disuse osteopenia. Based on experimental phenomena, we designed the basic approach of this study from the competitive mechanism of osteogenic and adipogenic differentiation, to explore how focal adhesion-related genes regulate the differentiation of mesenchymal stem cells in disuse osteoporosis. Identifying 8 key genes as potential regulatory target genes for the differentiation of mesenchymal stem cells into osteoblasts or adipocytes under the condition of disuse osteoporosis (ITGB3, LAMC1, COL6A3, ITGA8, PDGFRB, ITGA4, LAMB1, LAMA4). Three types of cells are potential target cells in disuse osteoporosis (chondroprogenitor cells, osteoprogenitor cells, and adipocyte progenitor cells). ITGA4, ITGA8, ITGB3 are members of the integrin family, which through non-covalent dimer formation, connect the extracellular matrix to the cytoskeleton to perform biological functions including mechanical signal transmission and guidance of cell differentiation (59). During the differentiation process of mesenchymal stem cells, the expression of integrins is dynamically regulated, for instance, during the differentiation of hMSCs, the expression of certain integrin subunits changes in correlation with the increase in osteogenic markers (60, 61). *In vitro* studies show that integrins also play an important role in intrachondral ossification, including but not limited to promoting osteogenic differentiation in hypoxic environments (62, 63). For example, ITGA4 is considered a rare early marker gene for cartilage, with chondrocytes expressing ITGA4 exhibiting more primitive characteristics compared to other cartilage cells (64). ITGA4 has been reported in the context of human articular cartilage lesions (65), with its expression being specifically upregulated in cells undergoing pathological degeneration compared to normal cells (66). ITGA8 can regulate the expression of integrins to induce cartilage formation, with experiments demonstrating that affecting ITGA8 expression during cartilage development leads to changes in the expression of ITGA4 and COL6A1 (67). Additionally, another study indicates that the subunit encoded by ITGA8 can activate Latent TGF-β (68), and the TGF-β signaling is highly activated in disuse osteoporosis, with inhibition of TGF-β signaling offering a potential rescue for the condition (69). Although ITGB3 has been reported to promote osteogenic differentiation (70), it is also noted that dysfunction of its function can be detrimental to the stability of cartilage and differentiation of chondrocytes (67, 71). Articular cartilage and growth plate cartilage both belong to hyaline cartilage, with their molecular mechanisms of formation and cell differentiation being similar (72, 73). The integrin-related genes we identified suggest the possible involvement of endochondral ossification during the process of long bone growth. This process may be disrupted in the context of disuse osteoporosis. Single-cell data analysis reveals that clusters of chondroprogenitor cells exhibit significant expression of these key genes (**Figure 7C**). This observation is corroborated by animal models using AB-PAS staining (**Figure 1B** AB-PAS staining), where, in mice with unloaded hindlimbs, a notable reduction and disappearance of osteogenic islands centered around hypertrophic chondrocytes are highlighted in the staining results. This marks our first report of the disruption in the development of growth plate cartilage under conditions of disuse osteoporosis.

Similar to the integrin family, the genes we screened include extracellular matrix components: LAMA4, LAMB1, LAMC1, COL6A1. These genes play crucial roles in the interaction between cells and the extracellular matrix, influencing various biological processes. LAMA4 is considered a negative regulator of adipocyte differentiation (74, 75), showing increased levels in both obese populations and mouse models of obesity (76, 77). The elevation in LAMA4 is thought to be a compensatory response due to the accumulation of fat droplets, which is significant in the context of disuse osteoporosis development. Associated with adipogenesis and obesity (78–82), LAMB1 is highly expressed during the mid-stage of adipogenesis (83). The LAMA4 expression facilitates the formation of fat droplets supported by the extracellular matrix. Upregulation in GSE100930 and qPCR results from animal models suggests that adipogenesis in stromal cells within the bone marrow cavity might be enhanced by extracellular matrix components. Similar to LAMA4 and LAMB1, LAMC1 is closely related to adipogenic differentiation, with significant overexpression in obesity-associated samples (84). Its role in promoting adipogenesis and the maturation of preadipocytes underscores the complex interaction between extracellular matrix components and adipocyte differentiation (85). As the only collagen-related gene screened, COL6A3 is also linked to adipogenesis (86). Its knockdown in 3T3-L1 cells led to a decreased fat breakdown function (87), highlighting its potential role in osteoporosis induced by steroid compounds (88). Interestingly, COL6A3 is also associated with insulin resistance (89), pointing to its significant role in metabolic diseases and wound healing challenges after long-term space missions (90, 91). The question of whether COL6A3 upregulation is a primary cause of insulin resistance leading to microgravity/disuse osteoporosis deserves further investigation. Distinct from the genes previously discussed, PDGFRB, a receptor gene, plays a unique role in influencing osteoporosis. Activation of PDGFR signaling has been found to enhance the proliferation, migration, and angiogenic capabilities of osteoprogenitor cells and stem cells, which supports bone repair processes (92). Specifically, boosting the PDGFR/Wnt/β-catenin signaling pathway in mesenchymal stem cells has demonstrated potential in mitigating osteoporosis in the OVX model (93). On the contrary, within the osteogenic lineage, PDGFR signaling elevates the expression of Csf1, which in turn promotes osteoclast development. The absence of PDGFRA/B in osteoblasts leads to a reduction in osteoclast numbers and an increase in trabecular bone volume, underscoring its therapeutic potential for osteoporosis (94). The question of whether elevated PDGFRB levels directly or indirectly contribute to the excessive bone resorption observed in disuse osteoporosis due to microgravity remains a subject for further exploration (**Figure 1B** TRAP staining).

In our key genes GSEA enrichment analysis, we primarily identified enrichment in signaling pathways such as “Cell Cycle,” “DNA Replication,” “Spliceosome,” and “Valine, Leucine, and Isoleucine Degradation” among various biological processes. Notably, among these genes, five key genes associated with adipogenesis (ITGA4, ITGA8, ITGB3, LAMA4, LAMB1) and three key genes in the osteogenesis process (ITGA8, ITGB3, COL6A3) were all enriched in the “Peroxisome” signaling pathway. This observation suggests that changes in peroxisome function may directly or indirectly influence bone mass changes during disuse osteoporosis. Peroxisomes are small organelles essential for maintaining cellular and metabolic equilibrium, redox metabolism, the oxidation of long-chain fatty acids, and the biosynthesis of ether lipids. Dysfunctions of peroxisomes are linked to various pathological states, declines in tissue functionality, and the aging process. Their contained special peroxidases, capable of reducing oxygen free radicals, help mitigate cellular oxidative stress and organelle damage (95, 96). Oxidative stress induced by microgravity or simulated microgravity conditions has been extensively documented (97–99), making antioxidation a potential strategy to alleviate disuse osteoporosis (100–103). Discussing the competitive mechanisms of adipogenesis and osteogenesis, the adipogenic differentiation of mesenchymal stromal cells is notably enhanced during disuse osteoporosis (**Figure 1C**), particularly with the increase in PPARG expression (**Figure 1C**). This indicates that enhanced adipogenesis is a significant factor in the occurrence of disuse osteoporosis. Considering the crucial role of Peroxisome Proliferator-Activated Receptors (PPARs) in regulating adipogenic and osteogenic differentiation (104, 105), Our GSEA enrichment analysis further suggests that modulating peroxisomal balance may be a feasible strategy under conditions of disuse osteoporosis. However, this remains a preliminary hypothesis based on the findings of this study, and the precise effects require further extensive experimental investigation. In the gene-gene interaction (GGI) network, we identified several genes beyond the integrin and laminin families. Among the key osteogenic genes, we observed genes related to osteoblasts or osteocytes, such as SPP1, IBSP, and DMP1 (106). Notably, NPNT has been reported to positively regulate the ERK1/2 signaling pathway, thereby promoting osteogenic differentiation. In contrast, among the genes interacting with key adipogenic factors, LPXN, TNC, THBS1, and OGG1 have been directly associated with obesity and fat accumulation (107–110). The construction of the GGI network led us to hypothesize that modulating key genes could influence both osteogenic and adipogenic processes, potentially affecting the progression of osteoporosis. The principal cause of osteoporosis is an imbalance between the processes of bone formation and resorption, which leads to a discrepancy between the rates of bone mineral deposition and dissolution. This imbalance culminates in a decrease in bone mass and a deterioration of bone microarchitecture, significantly elevating fracture risk (111). A focus on the differentiation of cells within the osteogenic lineage underscores the importance of examining the transcriptional profiles and differentiation behaviors of mesenchymal stem cells. In this light, leveraging single-cell sequencing data from mesenchymal cells (GSE147287) enables the identification of key genes that illuminate the characteristics of critical cell clusters involved in osteoporosis development (**Figure 7C**) and allows for the investigation of the expression patterns of these key genes within critical cells (**Figure 8D**). Our clustering and annotation identified nine cell subgroups, yet the key genes revealed in the integrative analysis were predominantly expressed in osteoprogenitor cells, adipoprogenitor cells, and chondroprogenitor cells (**Figure 8C**). This finding is expected, as the formation and differentiation of these precursor cells are fundamental to constructing the bone tissue’s extracellular matrix environment. Our analysis also highlights the impact of microgravity on osteogenesis, specifically its influence on extracellular matrix components, corroborating previously reported phenomena (112–114). GSEA analysis of key cell clusters in single-cell data revealed that pathways enriched in chondrocyte progenitors include “ribosome”, “MAPK signaling pathway”,” NF-κB signaling pathway”,”cytokine-cytokine receptor interaction” and “tumor necrosis factor signaling pathways”. In adipocyte progenitors, in addition to “ribosome” enrichment, pathways related to energy metabolism, such as “oxidative phosphorylation” and “thermogenesis” were highlighted. In osteoblast progenitors, we focused on pathways related to “osteoclast differentiation”, “tumor necrosis factor signaling” and “MAPK signaling pathway”. Interestingly, in the enrichment results for chondrocyte and osteoblast progenitors, the “osteoclast differentiation,” “MAPK signaling pathway,” and “TNF signaling pathway” were all commonly enriched. However, the enrichment scores for “osteoclast differentiation” and the “TNF signaling pathway” showed completely divergent results. This further emphasizes the cellular heterogeneity reflected in the single-cell data analysis results. (**Figure 7D**). Building on this, we used cell communication analysis to explore potential ligand-receptor interactions between osteogenic and adipogenic-related cells and other cell types. The results revealed that osteoblast progenitors exhibited a higher frequency of self-interactions. (**Figure 8A**).

Upon observing the key cell trajectories, we noted two branching events (**Figure 8B**), which clearly demonstrate two independent origins of osteogenesis. A large number of chondrocyte progenitors were observed to cluster near the right branch of Branch Point 1, a phenomenon that aligns with the initiation phase of endochondral ossification, particularly the proliferation and early differentiation of chondrocyte progenitors (115). Meanwhile, a significant number of osteoblast progenitors gathered at the upper branch of Branch Point 1, where osteoblast progenitors play a crucial role in the successful progression of intramembranous ossification (116). Based on these observations, we speculate that the right branch of Branch Point 1 may indicate endochondral ossification, while the upper branch likely suggests the occurrence of intramembranous ossification. The biological functions associated with branching point 2, as well as the related cell subgroups, merit further exploration and identification. The pseudotime analysis of cell trajectories revealed adipoprogenitor cells interspersed between chondroprogenitor and osteoprogenitor cells (**Figure 8C**). This analysis points to a competitive interaction during chondrocyte development and differentiation, echoing the findings of our key gene integrative analysis and supported by histological and morphological evidence (**Figure 1B** AB-PAS staining). The experimental results compel us to consider the issue of chondrocyte or hypertrophic chondrocyte transdifferentiation. Interestingly, the phenomenon of transdifferentiation of chondrocyte-related cells has also been reported through single-cell and transcriptomic analyses (117, 118). Furthermore, studies have shown that adipocytes can impair osteoblast function, and osteoblast progenitors can differentiate into adipocytes, which in turn reduces osteoblast function (119). Additionally, adipocyte progenitors can enhance adipocyte formation (120), potentially affecting bone cell function. In this study, we also observed that key genes were highly expressed in both adipocyte progenitors and osteoblast progenitors under microgravity conditions. This suggests that in environments lacking mechanical loading, key genes may influence adipocyte and osteoblast progenitors, thereby activating adipogenesis and potentially impacting bone health. However, further research is needed to confirm these findings. Finally, our pseudotime analysis of key gene expression revealed a general downward trend, with PDGFRB as an exception (**Figure 8D**).

Owing to constraints that precluded the performance of conditional knockout experiments to authenticate gene functionality, our research was limited to the identification of potential target genes and cells, complemented by the verification of these genes’ expression within animal models. The functionality of specific genes delineated in our project draws upon documented literature and inferential conjecture. We engaged in discussions on the gene and enrichment analysis results through lenses of universality and commonality. Literature aligned with our findings underscores disarray in the growth and differentiation of chondrocytes under disuse conditions. Staining techniques such as Alcian Blue-PAS indeed revealed disruptions in the differentiation and hypertrophy processes of growth plate chondrocytes. However, whether these findings are directly linked to the occurrence of disuse osteoporosis requires further discussion, as the closure of the growth plates in adults signifies the end of their participation in bone remodeling (121). Paradoxically, our analysis shifts the focus towards chondroprogenitor cells, intensifying the contradiction through animal model findings. This suggests that beyond the essential validation of key genes’ functions, there lies an urgent need to delve into the more complex biological roles of chondroprogenitor cells in adulthood, a revelation poised for future explorations.

Furthermore, by extracting primary bone marrow stromal cells from mice and conducting qPCR analyses, we observed significant changes in osteogenic and adipogenic differentiation-related genes in the hindlimb unloading (HLU) group. However, research on the specific expression patterns of these genes in well-established single-cell progenitor populations remains limited. We cannot directly determine the expression patterns of these genes across various cell populations, such as MALP, bone-mesenchymal stem cells, and early, mid, and late-stage progenitors. Therefore, future studies should further explore the expression differences of these genes in different progenitor cell populations and elucidate their potential roles in bone tissue formation and related diseases.

Additionally, there are some inconsistencies in this study, for example, we observed a consistent direction of gene expression changes in the HLU data, while the direction of changes was opposite in the GSE100930 data (**Table 1**). We believe these discrepancies may be related to differences in experimental conditions. Specifically, the HLU model simulates a microgravity environment through physical unloading, primarily focusing on the effects of mechanical unloading on bone metabolism. In contrast, the GSE100930 dataset originates from cell cultures in an actual microgravity environment, which likely involves more complex biological processes and signaling pathways. Due to these differences in experimental conditions, the observed changes in gene expression may differ. Another critical issue is that the method we used to obtain bone marrow stromal cells via a 3-day adherent culture for gene expression analysis makes it difficult to estimate the extent to which *in vitro* culture could influence the results. To address this, we plan to conduct further experiments using different models in future studies, such as employing a rotating cell culture system (RCCS) to simulate microgravity (16), in order to minimize these effects and enhance the generalizability of our findings. Lastly, while this study was conducted using a mouse model, there are still biological differences between mice and humans. For example, there is a significant difference in the timing of growth plate closure between adult humans and mice. Therefore, in future research, we plan to collect more clinical human samples of disuse osteoporosis to make our findings more clinically relevant and closer to practical applications.

## Data Availability

The raw data supporting the conclusions of this article will be made available by the authors, without undue reservation.
