# Supplementary material for: The potential key genes within focal adhesion that regulate mesenchymal stem cells osteogenesis or adipogenesis in microgravity related disuse osteoporosis: an integrated analysis
Source: Front Endocrinol (Lausanne). 2025 Mar 10;16:1469400. doi: 10.3389/fendo.2025.1469400 (PMC11930814; doi:10.3389/fendo.2025.1469400)
Supplement: Supplementary file 1 [file Presentation1.pdf]

Supplementary Material

1 Supplementary Figures and Tables

1.1 Supplementary Figures

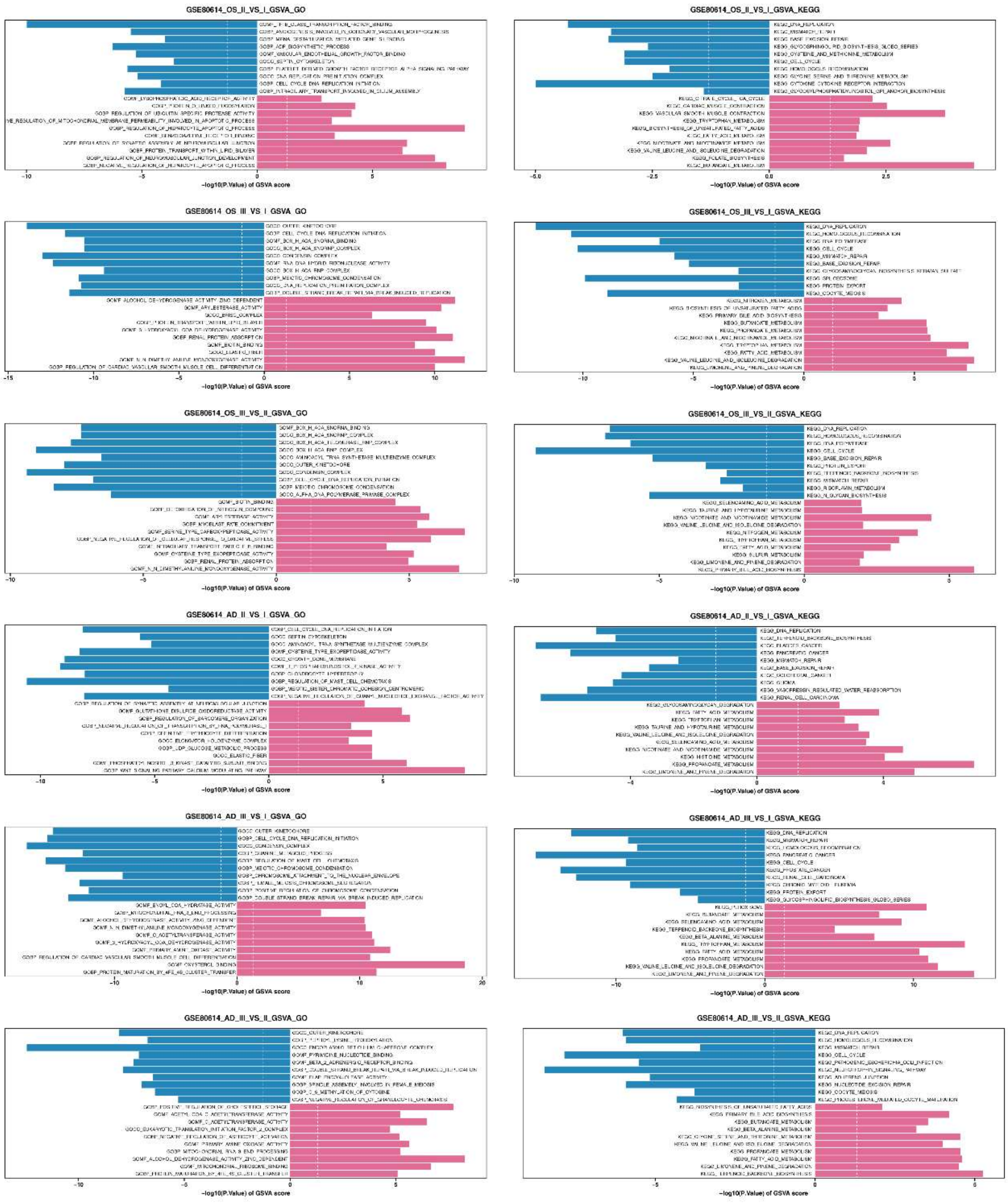

Supplementary Figure 1. GSVA enrichment analysis of 66 hMSC samples from three different differentiation stages (II vs I, III vs I, III vs II) during osteogenic and adipogenic differentiation.

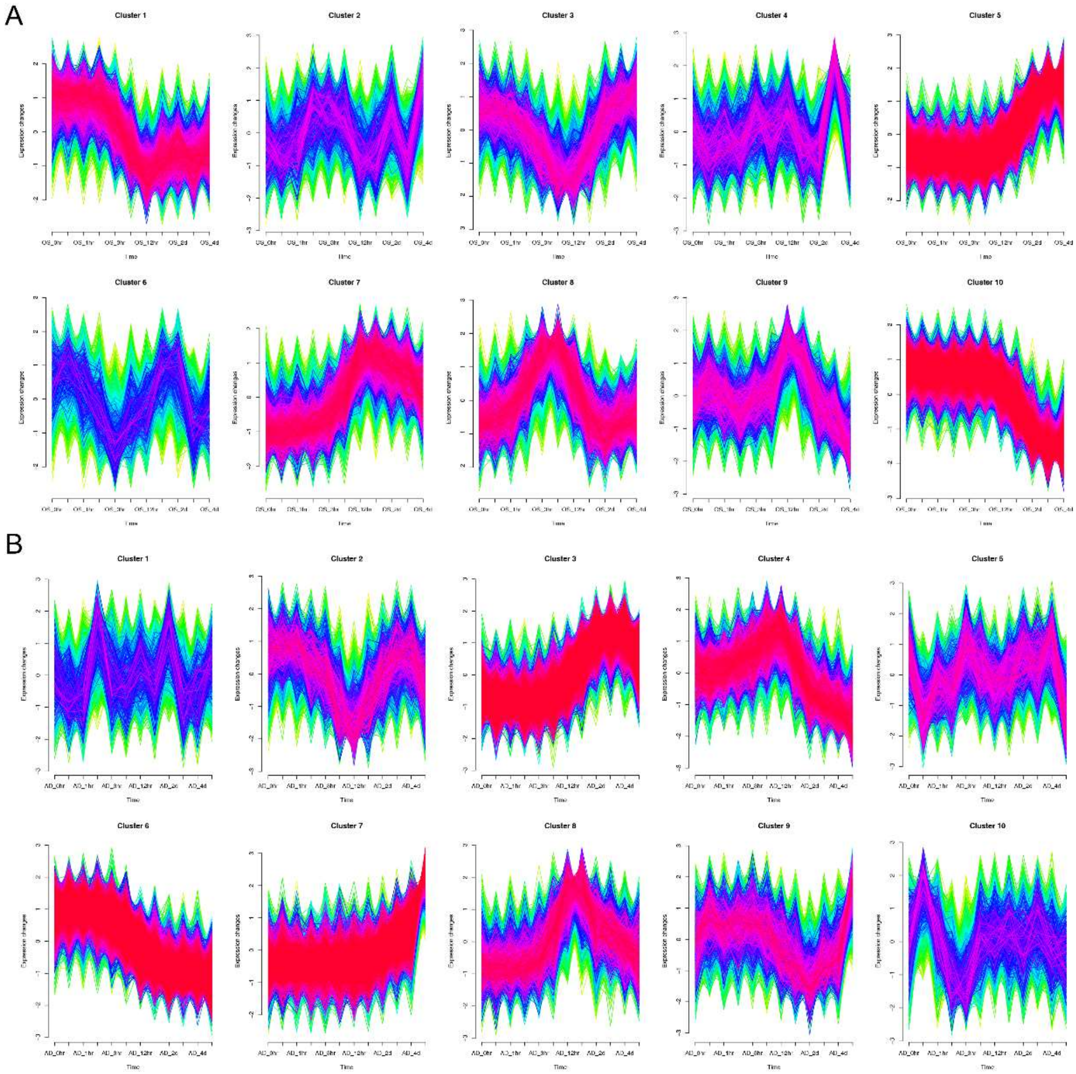

Supplementary Figure 2. (A) Soft clustering of osteogenic datasets. **(B)** Soft clustering of adipogenic datasets.

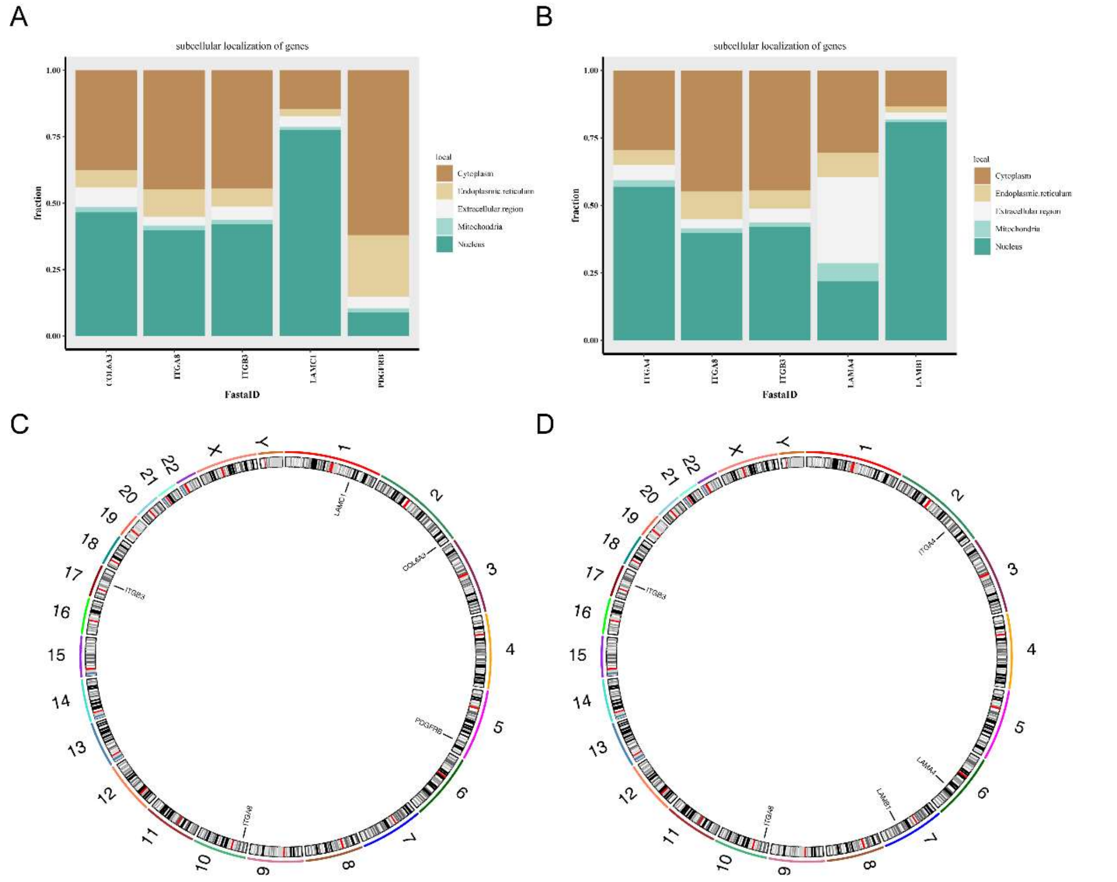

Supplementary Figure 3. Cellular and genetic localization in osteogenesis and adipogenesis. (A)Subcellular localization of osteogenic-associated cells. (B) Subcellular localization of adipogenicassociated cells. (C) Chromosomal localization of key genes for osteogenesis. (D) Chromosomal localization of key genes for adipogenic.

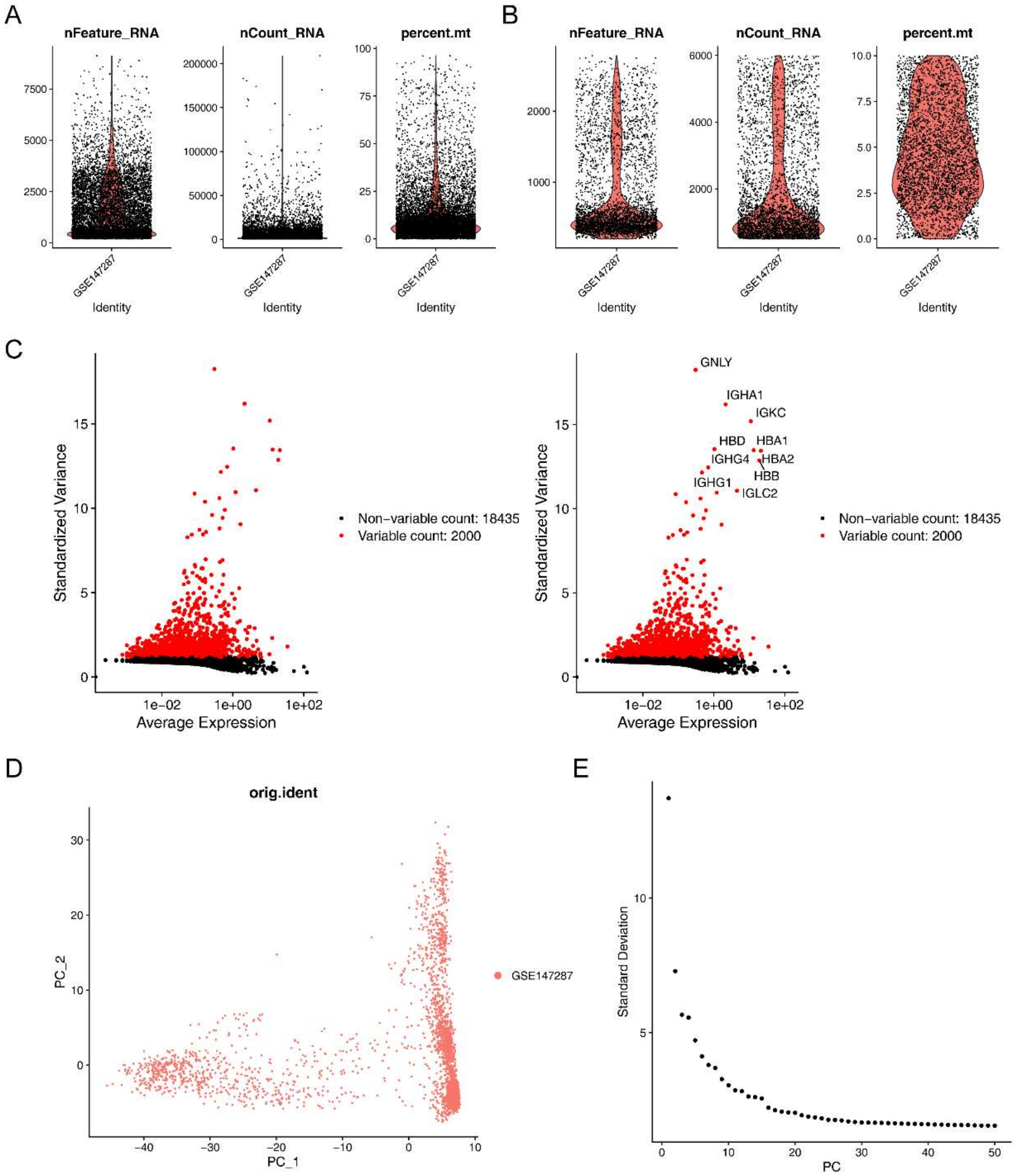

Supplementary Figure 3. Single-cell data analysis: filtering, gene variability screening, PCA evaluation, and key gene identification. (A-B) Results before and after filtering of single-cell data. (C)Screening of the first 2000 highly variable genes. (D) PCA replacement test and inflection diagram. (E) Top 9 PCA-related genes

1.2 Supplementary Tables

Supplementary Table 1. The list of qPCR primer sequence.
Supplementary Table 2. The information on 203 focal adhesion-related genes (FARGs).
Supplementary Table 3. KEGG enrichment analysis of 203 focal adhesion-related genes
Supplementary Table 4. MicroCT analysis results
